# In vitro evaluation of immunomodulatory, anti-diabetic, and anti-cancer molecular mechanisms of *Tribulus terrestris* extracts

**DOI:** 10.1038/s41598-022-26742-6

**Published:** 2022-12-28

**Authors:** Abdullah Khalid, Tariq Nadeem, Muhammad Asaf Khan, Qurban Ali, Muhammad Zubair

**Affiliations:** 1 Department of Bioinformatics and Biotechnology, Government College University Faisalabad, Faisalabad, Pakistan; 2 Centre of Excellence in Molecular Biology, University of the Punjab, Lahore, Pakistan; 3 Institute of Plant Breeding and Biotechnology, MNS-University of Agriculture, Multan, Pakistan; 4 Department of Plant Breeding and Genetics, Faculty of Agricultural Sciences, University of the Punjab Lahore, Lahore, Pakistan

**Keywords:** Cancer, Cancer prevention

## Abstract

Dampened immunity and impaired wound healing in diabetic patients may lead to diabetic foot ulcer disease, which is the leading cause of limb amputations and hospitalization. On the other hand, cancer is the most significant cause of mortality globally, accounting for over 10 million fatalities in 2020, or nearly one in every six deaths. Plants and herbs have been used to treat chronic diseases due to their essential pharmaceutical attributes, such as mitigating drug resistance, ameliorating systemic toxicities, reducing the need for synthetic chemotherapeutic agents,and strengthening the immune system. The present study has been designed to evaluate the effects of *Tribulus terrestris* on wound healing, cytotoxic and anti-inflammatory responses against HepG-2 liver cancer cell line. Two solvents (methanol and ethanol) were used for root extraction of *T. terrestris.* The wound healing potential of the extracts was studied on diabetic cell culture line by scratch assay. The anti-oxidant and cytotoxic potentials were evaluated by in vitro assays against HepG2 cell line. The methanolic root extract resulted in the coverage of robust radical scavenging or maximum inhibition of 66.72%,potent cytotoxic activity or reduced cell viability of 40.98%, and anti-diabetic activity having mighty α-glucosidase inhibition of 50.16% at a concentration of 80 μg/ml. Significant reduction in the levels of LDH leakage (56.38%), substantial ROS (48.45%) and SOD (72.13%) activities were recorededMoreover, gene expression analysis demonstrated the down-regulation of inflammatory markers (TNF-α, MMP-9, Bcl-2, and AFP) in HepG-2 cells when treated with *T.* *terresteris* methanolic extract as compared to stress. Furthermore, the down-regulation of inflammatory markers was validated through ELISA-mediated protein estimation of IL-1β and TNF-α. It is expected that this study will lay a foundation and lead to the development of efficient but low-cost, natural herbs extract-based dressing/ointment for diabetic patients and identify potential drug metabolites to treat out-of-whack inflammatory responses involved in cancer onset, progression, and metastasis.

## Introduction

Cancer and diabetes are the leading causes of death worldwide, and the development of novel anticancer and antidiabetic drugs has become the most pressing necessity in recent years^1^. Although there are different types of treatment, such as immunotherapy, chemotherapy, radiation, and hormonal therapy, they all possess substantial shortcomings, including severe toxicities and drug resistance^2^. The limitations of targeted therapy become apparent after years in practice. The vast majority of malignancies are not targetable, because they evolve adaptive mechanisms and multiple oncogenic pathways^3^.

Therefore, the limitations mentioned above drive up the demand for plant-based natural products possessing therapeutic efficacy for diseases ranging from the common cold to cancer and diabetes^4,5^. Herbal remedies rarely cause serious adverse effects, and reports of deaths or hospitalizations as a result of these remedies are exceptionally rare. The United States National Poison Control Center does not even have a section for herb-related adverse effects in its database. The healing process can be generally divided into four phases: the hemostatic, inflammatory, proliferative, andremodeling phase, ultimatelydetermining the strength and manifestation of the healing tissue^6^.

The genus *Tribulus*, belonging to the Zygophyllaceae family, contains about 20 different species, of which three species *Tribuluscistoides*, *Tribulusalatus*, and *Tribulusterrestris*, are widely found in India^7^. *T.terrestris* extract has been shown to inhibit cell proliferation and promote apoptosis in liver cancer cells. Its biological activity has been related to steroidal saponinsby inducing an apoptotic pathway in breast cancer cells. Trans-N-feruloyl-3-hydroxytyramine and trans-N-feruloyl-3-ethoxytyramine, have been shown to trigger apoptosis in leukemic cancer cells^8–10^.

Plant anti-oxidant compounds are commonly isolated by solvent extraction technique. However, the extract yields and anti-oxidant activities of the plant materials are mainly dependent on the nature of the solvent, polarities, and presence of various anti-oxidant compounds of diverse characteristics of chemical which may or may not be soluble in a specific solvent^11,12^. Polar solvents are normally used to recover polyphenols from the plant material. The most appropriate of these solvents are aqueous mixtures in Methanol, ethanol, acetone, and ethyl acetate. Ethanol and Methanol have been widely used to extract anti-oxidant compounds from plants and plant-based foods like plum, pomegranate, rosemary, rice bran, wheat grain, citrus peel, and many other fruit peels^13,14^.

*Tribulusterrestris* extract containsalkaloids, resins, flavonoid oil, and nitrates, which have antihypertensive, anti-inflammatory, hypolipidemic, and anti-diabetic activities^15^. The ethanolicextract of *T. terrestris* has been reported topossesssubstantial anti-oxidant activity against STZ-induced diabetic rats, significantly reducingout-of-whack serum glucose,triglycerides, and cholesterol levels. Furthermore,serum superoxide dismutase (SOD) activity was enhanced in alloxan-induced diabetic mice with subsequent inhibition of gluconeogenesis^16–18^. To evaluate the effectiveness of *T. terresteris* plant extract, we studied the anti-diabetic, cytotoxic, and anti-inflammatory properties of methanol and ethanol solvent-based extracts. We used HepG-2 liver cancer cell line to study the cytotoxic activity response of root extract.

## Materials and methods

### Extract preparation

Fresh samples of the genotype of *T. terrestris* were obtained in April 2019 from the University of Agriculture, Faisalabad, Pakistan. It has been confirmed that the experimental sample plants (*T. terrestris*), including the collection of plants, complied with relevant institutional, national, and international guidelines and legislation with appropriate permission from the Department of Bioinformatics and Biotechnology, Government College University Faisalabad, Pakistan. A specimen voucher was put forward to the herbarium department. Roots were washed, dried at 37 °C, and ground to get a fine powder. Ethanol and methanol solvents were used for extraction purposes. These solutions were placed in an incubator for two days for shaking purpose. Each solution was filtered with Whatman No. 1 filter paper. The solvents were evaporated with the help of a rotary evaporator, and extracts were dissolved in phosphate buffer saline (PBS) for use in future studies.

### Antidiabetic activity: α-glucosidase assay

The anti-diabetic activity of *Tribulus terrestris* (ethanol and methanol-based extracts of the samples dried at 37˚C) was assessed using an α-glucosidase assay. A reaction mixture having 12.5 μL of a *T. terrestris* extract, 40 μL of α-glucosidase (5μ/mL),and 140 μL of phosphate buffer saline 1X (pH 7.4) was added to 96-well plates. Incubation was done at 37˚C for 5 min, and 40 μL 4-nitrophenyl-β-D glucopyranoside (PNPG 5 mM) was added to each well. Acarbose was used as a positive control with aconcentration of 3 mg/ml and PBS as a negative control. An ELISA plate reader was used to measure optical density at 405 nm. Percent inhibition of α-glucosidase was calculated as [(Ac–As)/(Ac)]*100, where Ac and As shows the absorbance of the control and sample, respectively.

### Wound healing: scratch assay

The retinal pigment epithelial (RPE) cell line was cultured at the rate of 2 × 105 cell/ ml in Dulbecco’s modifiedEagle’s medium (DMEM) containing 10% fetal bovine serum (FBS) and 1% penicillin/streptomycin for 24 h on 12- well cell culture plates at 37 °C and 5% CO2 in an incubator to produce 80–100% convergent cell monolayer. A sterile pipette tip of 200 μL was used to create a linear wound. The wells were washed two times with PBS by removing the medium. Each well was added with fresh medium containing ethanol and methanol-based extracts of *Tribulus terrestris*. DMEM containing 1 ml with 20 μLof PBS was used asa negative control. The platelet-derived growth factor (PDGF) at the rate of 5 ng/ml has been used as a positive control. Digital images of the wound on the cell monolayer wound were taken at 0 h, 4 h, 8 h and 16 h. Evaluation of these images at 1712 × 1368 pixels in Image J 1.440 software. The measureof wound healing from time to time compared with the initial width at 0 h to determine the percentage of cell migration and increase induced by the secretion of *Tribulusterrestris* extracts.

### Scratch assay

The assessment of migration potential of*T. terrestris* extract in RPE was tested by cell scratch assay. In short, 8 × 104 cells were cultured on a 6-well plate and left to form a monolayer. The monolayer was then scratched using sterile 200μLpipette tips after 80% confluency. The cells were then washed to remove the non-adhesive cells, and the medium was replaced. The images were taken in a phase contrast microscope abruptly after the scratch creation, followed by microscopy after 4 h, 8 h, 16 h, and 18 h.

### Cytotoxic activity: MTT assay

Cytotoxicity was assessed using human liver cancer cell line HepG2 cell line using *T. terrestris* extracts. The same medium described above was used in 96-well culture plates with an initial concentration of 2 × 104 cells per well. Each cell culture plate was treated with ethanol and methanol solvent-based *T. terrestris* extracts with a concentration of 0.1 mg/ml. Phosphate buffer saline (PBS) was used for negative control. After 2 days of incubation, 10 μL of MTT (3-(4,5-dimethylthiazol-2-yl)-2,5-diphenyltetrazolium bromide) was added to each well in a final concentration of 5 mg/ml. These plates were again incubated for 4 h. Optical density was measured at 490 nm after adding 150 μLof 0.1% dimethyl sulfoxide (DMSO). In the case of HepG2, percent inhibition was calculated as [(Ac–As)/(Ac)] *100, where Ac and As show the absorbance of the control and sample, respectively.

### Anti-oxidant activity determination

The plant extract was also assessed for anti-oxidant activity through DPPH assay. The DPPH (2,2-diphenyl-1-picryl-hydrazyl-hydrate) assay was used to determine the free radical scavenging activity released by *T. terrestris* extracts in ethanol and methanol solvents at various concentrations (5 μg/ml,10 μg/ml,20 μg/ml,40 μg/ml,80 μg/ml and 160 μg/ml). The stock solution (0.3 mM) DPPH was prepared in Methanol and ethanol with 10 µL of root sample was assorted with 190 µL of DPPH in 96 well plate or gallic acid taken as standard with consequent incubation period of 30 min in dark. Microplate reader (ELx808IU Biotek USA) was used to measure absorbance value at 517 nm and percentage inhibition of DPPH solution was calculated by their measurements.

$$ {\text{Percentage inhibition }} = \, \left( {{\text{control Absorbance}} - {\text{ sample Absorbance}}} \right)/{\text{ control Absorbance }}*{1}00. $$

### Cytotoxicity assessment through LDH assay.

LDH activity in media samples was measured as stated by the manufacturer's instructions (Roche Diagnostics, Cat No. 04744926001). In short, a mixture of lactate dehydrogenase was prepared by adding the same volume of LDH co-factor, dye, and substrate solution. Approximately 50 μL of media samples and 100 µL of LDH test compounds were added to each well in a 96-well plate. The incubation period of the plate was 20–30 min at room temperature. The stop solution was used to stop the reaction, and a microtiter plate reader measured the measurement of absorption at 490 nm and a reference absorption at 690 nm.

### Superoxide dismutase (SOD) assay

As the manufacturer's guidelines directed, the SOD assay kit determined the SOD activity (Abcam USA, Cat No. ab65354). The addition of 20 μL/well supernatant of different groups was followed by 200 μL of WST solution in each well, which included blanks 1, 2, and 3. The working solution of an enzyme (20 μL) was added to all wells of the samples and blank 1. Slightly stirred the plate for mixing, and the incubation period was 20 min at 37 °C. The absorption was measured at 450 nm using a microtiter plate reader.

### Determination of ROS generated by HepG2 cells

The ROS assay determined the number of reactive oxygen species produced by cells. The cell capability can be decreased, which enhances oxidative stress damage by increasing ROS production. ROS activity was measured with the help of the Cellular ROS Detection Assay Kit (Abcam, ab113851DCFDA) through the manufacturer's protocol. HepG2 cells (25 × 104) were added to 96 well plate at the p3 stage. The cells were washed three times with 1X PBS and 100 μLof DCF-DA working solution for 1 h at 37 °C. DCF-DA media was taken out, and cells were washed with 1X PBS. In each well, PBS 100 μL was added, and a spectrophotometer was used for fluorescence readings at 485 nm and 535 nm.

### Estimation of inflammatory cytokines concentration through ELISA

According to the manufacturer's protocol, the concentration of IL-1β and TNF-α was determined by using an ELISA kit. The cells were cultured at a density of 1 × 104 cells/well into 96-well plates for 24 h at 37 °C and then exposed to a methanolic extract of *Tribulus terrestris*. Untreated cells were used as a negative control. The collected material was tested with *IL-1β* (Invitrogen ELISA kit Catalog # **BMS224-2)** and TNF-α (Invitrogen ELISA kit Catalog # **KHC3011)** as per the protocol recommendations. A microplate ELISA reader determined the final absorbance at 405 nm.

### Real-time polymerase chain reaction for gene expression analysis

The trizol technique was employed to purify total RNA from the control, stress, and methanolic plant extract-treated HepG-2 cell line groups. A Nanodrop spectrophotometer was used to estimate extracted RNA. RNA was reverse transcribed into cDNA by RevertAidTM first-strand synthesis kit (qRT-PCR) according to the manufacturer’s instructions. The expression of the identified genes involved in HCC was investigated, and qPCR was performed using a generated cDNA template. Table 1 highlights the sequence of the primers. All real-time PCR tests were performed in triplicate, and GAPDH mRNA levels were measured to normalize the AFP, MMP-9, TNF alpha, and Bcl-2 mRNA expression values.

**Table 1:** Sequence of the primers.

Genes	Forward Primer	Reverse Primer	Reference
AFP	GCAGAGGAGATGTGCTGGATTG	CGTGGTCAGTTTGCAGCATTCTG	www.origene.com
MMP-9	GCCACTACTGTGCCTTTGAGTC	CCCTCAGAGAATCGCCAGTACT	www.origene.com
TNF-**α**	CTCTTCTGCCTGCTGCACTTTG	ATGGGCTACAGGCTTGTCACTC	www.origene.com
Bcl-2	ATCGCCCTGTGGATGACTGAGT	GCCAGGAGAAATCAAACAGAGGC	www.origene.com

### Statistical analyses

All experiments were performed in triplicates (n = 3), and data were expressed as mean ± standard deviation. Statistical analysiswas carried out using Minitab 16 software. Analysis of variance and Tukey’s post hoc tests were performed to compare different treatment groups. Graphs were plotted to show percentage scratch coverage after different treatments for the scratch assay data. Graphs were made through GraphPad Prism software (version 5.00 for Windows, GraphPad Software, USA).One-way ANOVA with Bonferroni’s test for significant differences between the groups was determined, and the p-value less than 0.05 was considered statistically significant.

## Results

### Anti-diabetic activity

**Figure 1:**
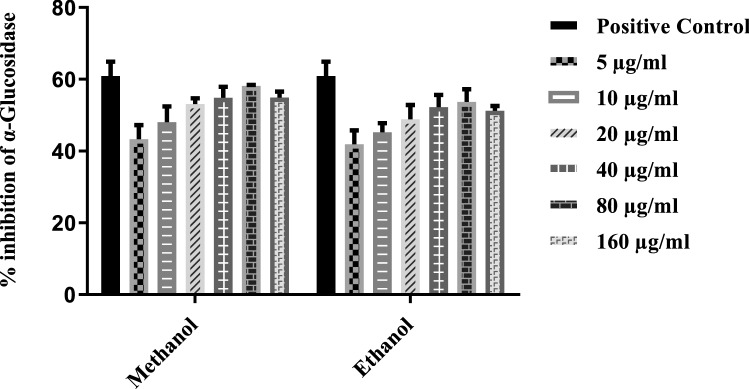
Effects of *T. terrestris* root extracts on α-glucosidase inhibition. Plant part root of *T. terrestris*were prepared in two different solvents. Extracts were tested at a concentration of (5 μg/ml, 10 μg/ml, 20 μg/ml, 40 μg/ml, 80 μg/ml and 160 μg/ml). Phosphate buffer saline was used as negative control. Acarbose at a concentration of 3 mg/ml was used as a positive control. Bars represent the mean (based on 3 replications of each treatment).

The protective effect against diabetes was evaluated by quantifying the inhibition of α-glucosidase using plant root extracts of *T. terrestris* (Fig. 1). Ethanol and Methanol were used for comparing potent solvent activity at various concentrations (5 μg/ml, 10 μg/ml, 20 μg/ml, 40 μg/ml, 80 μg/ml and 160 μg/ml). Methanol showed the best activity as compared to ethanol. Solvent exhibited a significant effect (*P* < 0.0001). Root extracts demonstrated the maximum inhibition activity of 58.16% at the concentration of 80 µg/ml dissolved in methanol solvent at 37 °C and minimum inhibition activity of 41.87% at 5 µg/ml when compared with negative control PBS. Acarbose used as a standard drug produced only 60.91% inhibition.

### Wound healing

**Figure 2:**
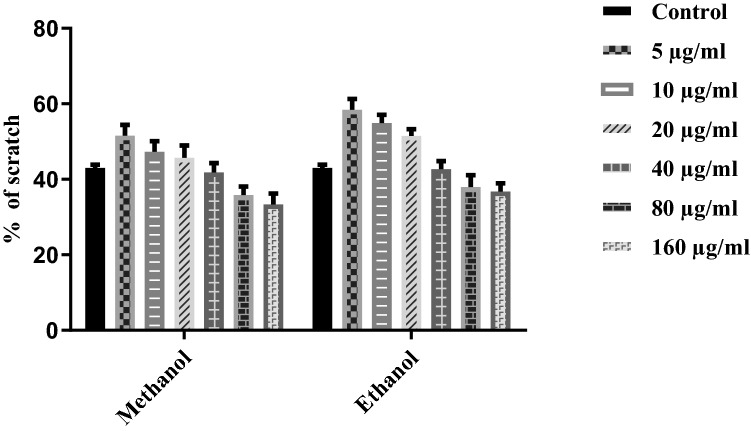
Effects of *T. terrestris* extracts of root on cell proliferation of retinal pigment epithelial (RPE) cells. *T. terrestris* extracts were prepared in two different solvents (ethanol and Methanol) and dried at 37 °C temperature and. Phosphate buffer saline was used as negative control and PDGF was used as positive control. Evaluations were conducted after 16 h of incubation. Bars showed the mean ± standard deviation (based on 3 replications of each treatment and 100 observations of scratch width for each replication).

**Figure 3:**
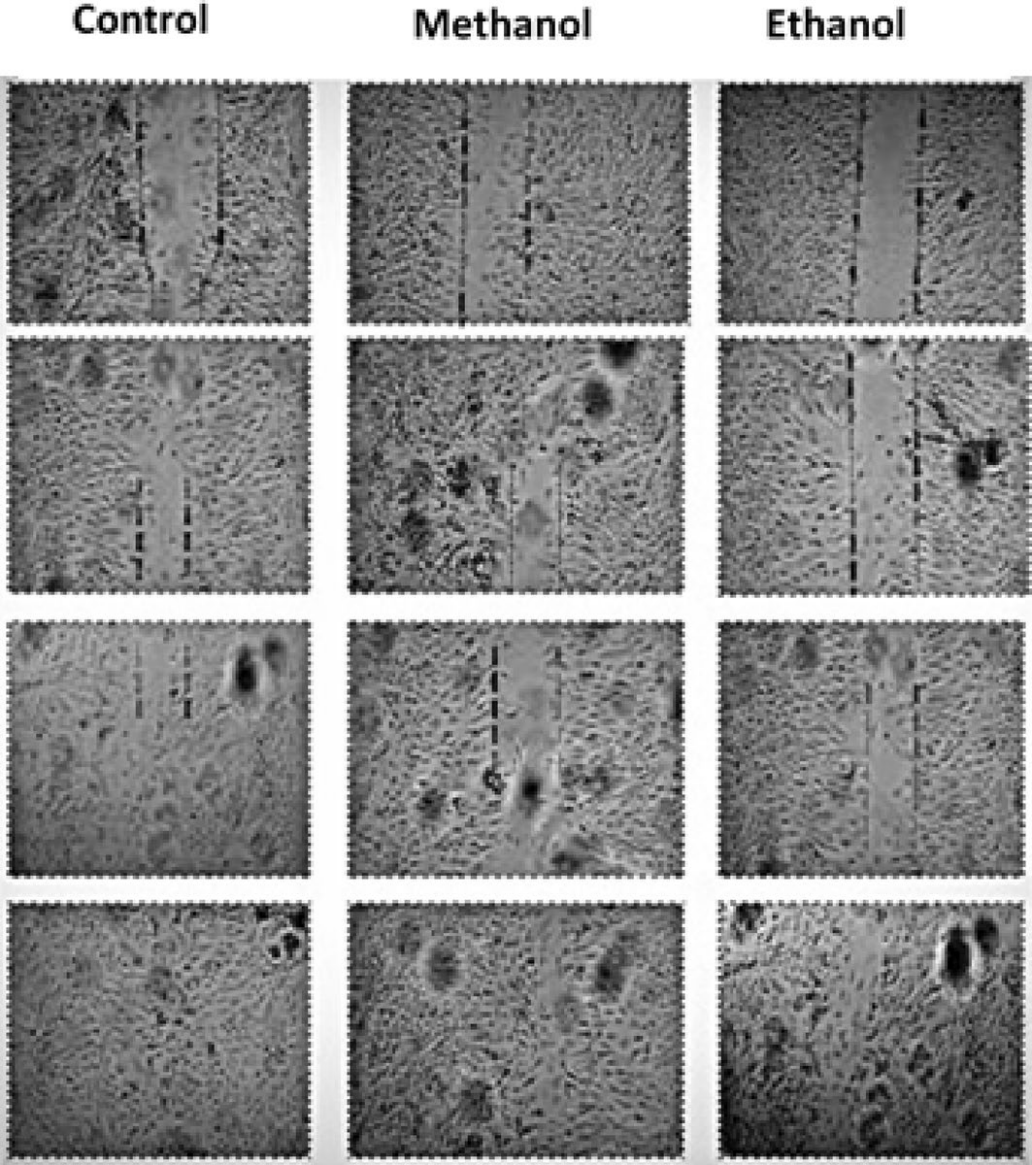
Effects of *T. terrestris* extracts of root on cell proliferation of retinal pigment epithelial (RPE) cells. *T. terrestris* extracts were prepared in two different solvents (ethanol and Methanol) and dried at 37 °C temperature and. Migration of cells have been noticed after 0, 4, 8 and 16 h of incubation.

Retinal epithelial cells were used in a cell scratch assay to study *T. terrestris* extract for the proliferation or migration of in vitro cells. The cells treated with *T. Terrestris* extracts covered the scratch area more rapidly than the cells of negative control, but even the methanolic extracts was more efficacious than the PDGF, which was the positive control. Tukey’s post hoc test and variance analysis were conducted independently, illuminating significant differences (*P* < 0.05) among the tested extracts. The scratch area expresses the data as a percentage (Figs. 2 and 3). There is a decrease in the percentage of scratch area with an increase in the concentration of *T. terrestris* extract.

### Cytotoxic activity

**Figure 4:**
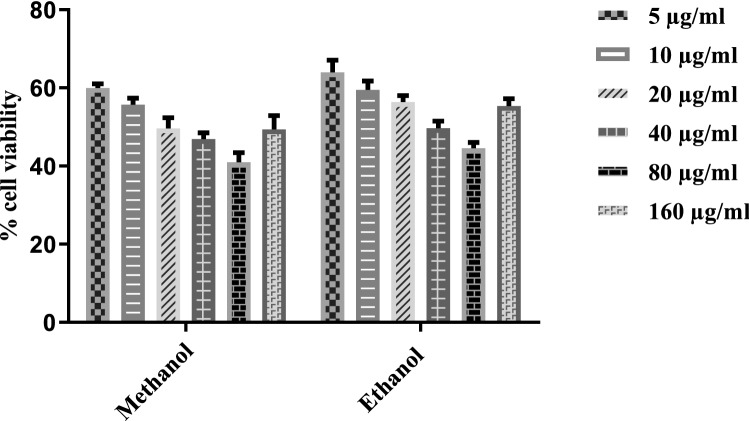
Effects of *T. terrestris* extract on HepG2 cell line.. *T. terrestris* extracts were prepared in two different solvents (ethanol and Methanol) and dried at 37 °C temperature and. Migration of cells have been noticed after 0, 4, 8 and 16 h of incubation. Extracts were tested at a concentration of 0.1 mg/mL. (5 μg/ml, 10 μg/ml, 20 μg/ml, 40 μg/ml, 80 μg/ml and 160 μg/ml). Bars represent the mean values (based on 3 replications of each treatment).

The cytotoxic activity activity of *T. terrestris* extracts against the HepG-2 cell line was assessed after 48 h. All the tested extracts showed significant cytotoxic activity activity compared with the control (Fig. 4). MThe methanol-based root extract showed reduced cell viability of liver cancer cells (40.98%) at the concentration of 80 μg/ml.M Extracts in the case of Methanol showed more inhibition and followed the same trend as ethanol but in a more potent manner.

### Anti-oxidant activity

**Figure 5:**
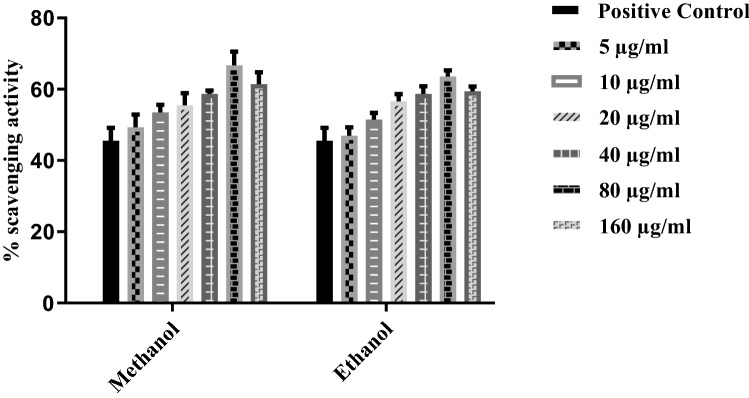
DPPH radical scavenging activity of root extracts in ethanol and zethanol of *T. terrestris*after 30 min of incubation.Bars with different alphabets indicate significant differences between treatment means at *p* < 0.05 based on Tukey’s post hoc test.

A comparison of root extracts of plants in two different solvents, i.e., ethanol and Methanol, after 30 min of incubation showed anti-oxidant activity at different concentrations (5 μg/ml, 10 μg/ml, 20 μg/ml, 40 μg/ml, 80 μg/ml and 160 μg/ml) (Fig. 5). Gallic acid was used as positive control. Methanolic extract showed maximum inhibition of 66.72% at 80 μg/ml concentration compared to ethanol, which was 63.56%. Scavenging activity increases with concentration but decreases after 80 μg/ml.

### LDH assay

**Figure 6:**
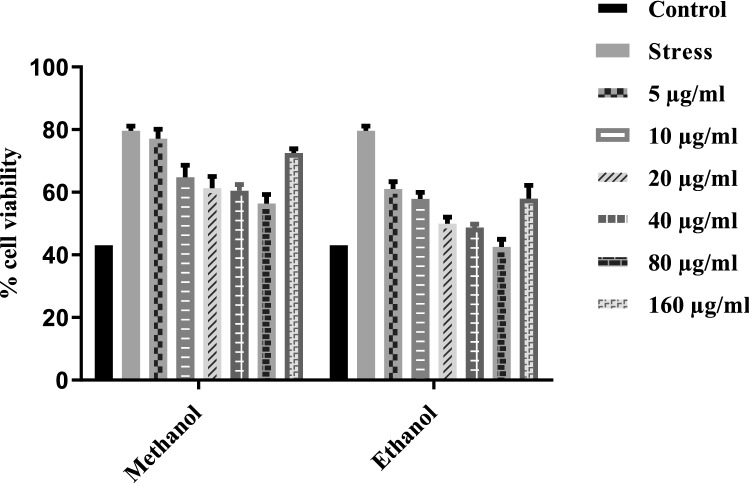
Cytotoxicity decreased by preconditioning of *T.terrestris*. The release of LDH is directly proportional to the impairment of the plasma membrane and high quantity damage/death the cell membrane. However, drastically low LDH was noticed in IL-1β induced HepG2 preconditioned with 40 μg/ml and 60 μg/ml T.T concentrations as compared to stress control.

The release of LDH is directly proportional to the impairment of the plasma membrane and the high quantity of damage/death to the cell membrane. However, drastically low LDH was noticed in IL-1β-induced Hep2 liver cancer cells preconditioned with *T. terrestris* extract concentrations compared to stress (Fig. 6).

### Superoxide dismutase assay

**Figure 7:**
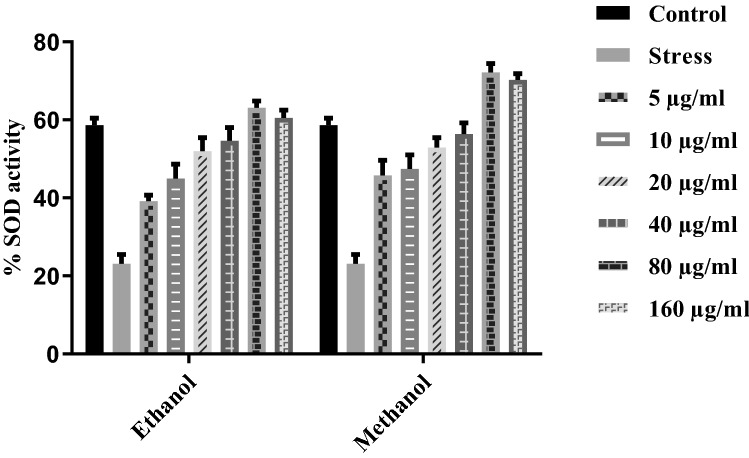
Superoxide Dismutase (SOD) is significant anti-oxidative enzyme present in cells that can reduce superoxide ions into less detrimental products. SOD activity is higher in normal cells compared to cells that respond to oxidative stress. We have seen higher SOD activity in *T. terrestris* preconditioned HepG2 when compared with IL-1β stimulate HepG2.

Superoxide Dismutase (SOD) is a critical anti-oxidative enzyme in cells that can reduce superoxide ions into less detrimental products (Fig. 7). SOD activity is higher in normal cells than in cells that respond to oxidative stress. We observed higher SOD activity in *T. terrestris* preconditioned HepG2 cells compared with IL-1β induced stress group.

### Reactive oxygen species assay

**Figure 8:**
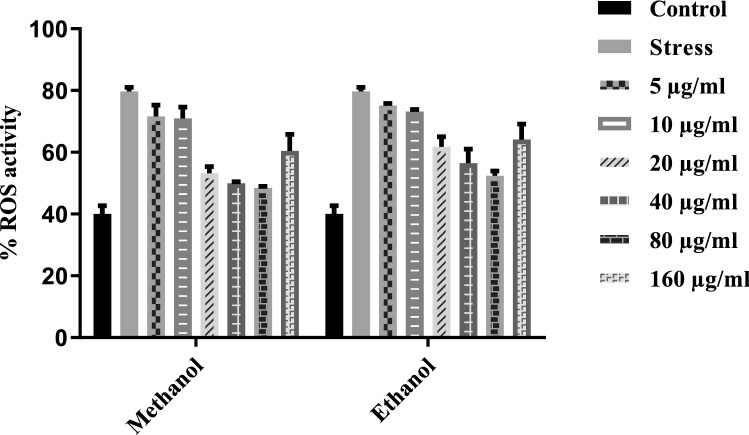
T.T preconditioned enhance the scavenging capacity of reactive oxygen species. Results showed that the cells preconditioned with 40 μg/ml and 60 μg/ml *T.terrestris* concentrations help in decreasing reactive oxygen species in IL-1β induced HepG2 cells as compared to stress condition.

The ROS assay determined the percentage of reactive oxygen species produced by cells. The cell capability decreases with enhanced oxidative stress damage due to increased ROS production (Fig. 8). ROS activity was measured with the help of the Cellular ROS Detection Assay Kit (Abcam, ab113851DCFDA) according to the manufacturer's protocol. HepG2 cells at the rate of 25 × 104 were seeded to a 96-well plate at the p3 stage. The cells were washed three times with 1X PBS and 100 μl of DCF-DA working solution, which was used for the staining for 1 h at 37 °C. The DCF-DA media was removed, and the cells were washed with 1X PBS. A spectrophotometer was used for fluorescence readings at 485 nm and 535 nm.

### Protein concentration studies of inflammatory cytokines through ELISA

Two different concentrations (20 μg/ml and 40 μg/ml) of *Tribulusterrestris* methanolic plant extract were subjected to assess the protein concentration of TNF-α and IL-1β through ELISA in the HepG-2 liver cancer cell line (Fig. 10A,B). The ELISA result shows that the methanolic plant extract decreases the protein concentration of IL-1 β at a 40 μg/mL concentration. Contrary to this, TNF-α protein expression increases when exposed to 40ug/ml methanolic extract compared to 20 μg/ml.

### Downregulation of inflammatory marker genes by methanolic plant extract in HepG-2 liver cancer cells

**Figure 9:**
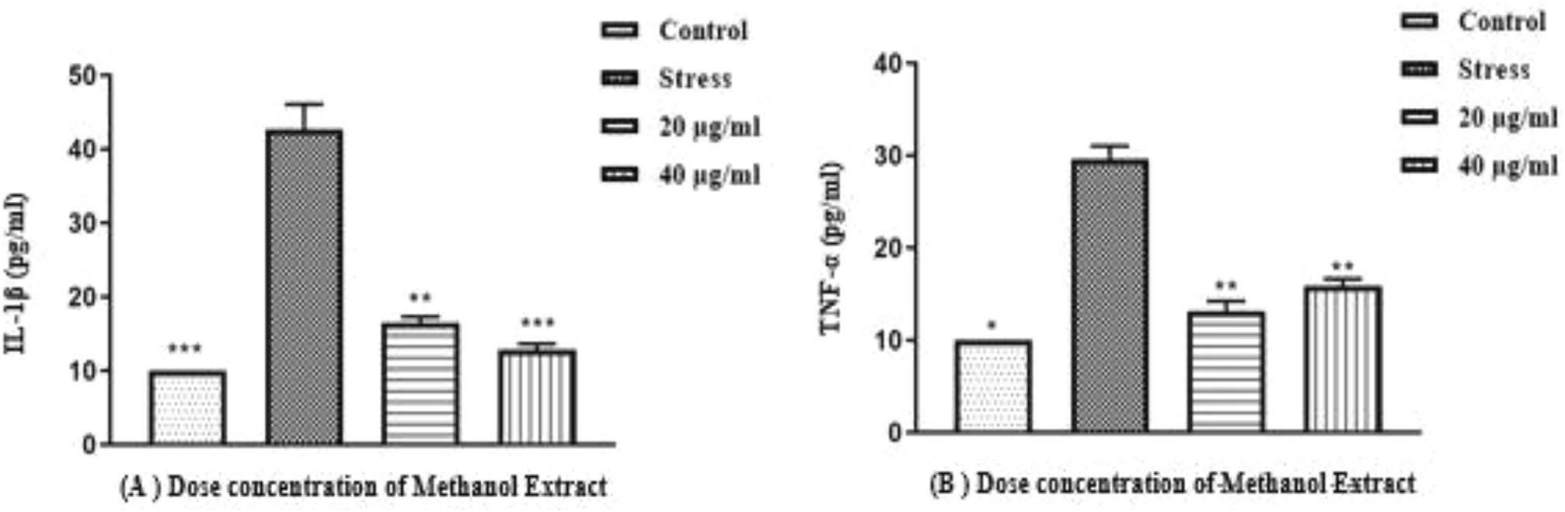
Cytokine concentrations (n = 3) of control, stress and methanolic plant extract treated HepG-2 cells (**a**) indicates protein concentration of IL-1β (**b**) represents the protein concentration of TNF-**α.** Bars represent standard deviation. Moreover, ∗ , ∗  ∗ and ∗  ∗  ∗ indicate the significance level at *p* < 0.5, *p* < 0:01, *p* < and *p* < 0:001.

In order to evaluate the mRNA expression levels of inflammation and apoptosis-associated genes, including tumor necrosis factor-alpha (TNF-**α**), matric metalloproteinase-9 (MMP-9), alpha-fetoprotein (AFP), and B-cell leukemia and lymphoma 2 (Bcl-2), RT-PCR study was performedusing *T.terrestris*methanolic extract treated HepG2 cells line along with GAPDH, which was tested as a housekeeping gene. Methanolic plant extract of *T. terrestris* significantly downregulated the mRNA expression of anti-apoptotic and inflammatory genes in the liver cancer cell line. Figure 9 shows the expression variation of TNF-**α**, MMP-9, AFP, and Bcl-2. Methanolic plant extract of *Tribulusterrestris* diminished the expression of tumor-mediated genes in a dose-dependent manner, particularly at a concentration of 20 μg/ml.

## Discussion

The concentration of bioactive compounds in plant extract is affected by factors like drying conditions, extraction protocol, chemical nature, storage conditions, and duration. The solvent used in the extraction of plant material plays a vital role in the recovery of bioactive compounds, which affects the health benefits^19,20^. Methanol and ethanol are more commonly used solvents; their boiling points are 64 ℃ and 78.4 ℃, respectively. Therefore, in the case of Methanol, there is a low-temperature requirement to evaporate the solvent in the rotavapor, so there is less harm to plant extract metabolites than ethanol. In literature, most polar compounds like phenolics, alkaloids, and terpenes have shown potent anti-diabetic and cytotoxic activity activity when dissolved in methanol and ethanol solvents^13,21,22^.

Inhibition of α-glucosidase activity was studied by adding *T. terrestris* extracts dissolved in ethanol and Methanol. We tested the extracts prepared in two solvents (Methanol and ethanol) to check the enzymatic inhibitory response against α-glucosidase (Fig. 1). MM Methanol-based root extract showed robust activity at 80 μg/ml concentration compared to ethanol.ExtractiMng polyphenols using solvents like Methanol, ethanol, and acetone are more proficient alternatives than organic solvents^23^. Every solvent has a unique property that may vary among solvents. While on the other hand, ethanol and Methanol are more impactful for extracting polyphenols from *Hibiscus sabdariffa*^24^. According to our results, Methanol provided the highest extraction yield. Polarity and chemical nature may be the source of variation in phenolic compounds in different organic solvents. In the case of organic solvents ethanol and Methanol, the addition of water creates a more polar medium which can enhance the yield of phenolic compounds even more, but the maximum concentration of polyphenols was gained by using pure solvents from the *C. citratus*^25^*.*

The cells treated with the *T. Terrestris* extracts covered the scratch area more quickly than the negative control cells. Even the best extracts were more proficient than the positive control. Coverage of scratch area by cell proliferation expresses the data as a percentage. Root showed covering of scratch area maximum in case of Methanol compared to ethanol (Figs. 2 and 3). In the case of wound healing, the essential stage is cell proliferation, and a variety of plant species have been found to study the coverage of wound healing by using the scratch test^26^. Our research showed magnificent results on wound healing by migration and proliferation of cells by using the extract of *T. terrestris* at a rapid rate, even higher than any positive control. In earlier studies, the optimal time after adding extracts treatment was 12–72 h to estimate the cell proliferation, though the time of cell proliferation may vary depending on the cell type, plant extract, and width of scratch^27^.

In the present study, the optimum time for cell proliferation was 0–16 h after treating cells with the extract. Moreover, roots used for the Mmethanol extraction had meaningful conclusive results on cell proliferation. A compelling study of cell proliferation of freeze-dry leaves of *Plantago major* showed that the optimum concentration was 1.0 mg/mL on a dry weight basis, and in contrast, 0.1 mg/mL was better than 10 mg/mL^28^. It is also noted that ethanol-based extracts with a higher concentration (10 mg/mL)had a deleterious impact on cell multiplication and migration. In our research, concentration was confined to 0.1 mg/mL in different parts of plant extracts. Plant extracts with the most optimum concentration of particular compounds may obstruct or retard cell migration and proliferation^29^. On the other hand, extract taken from the plant of legume genus *Astragalus* and *Simmondsia* jojoba seeds (jojoba liquid wax; 5% v/v) induced slow migration and proliferation of cells, while compared to the extracts of lower concentration (1 ng/mL and 1%v/v) respectively^30^.

The investigation of medicinal plants for the potential cytotoxic activity effect is assessed by evaluating their ability to neutralize cancer cells in vitro. In the present study, extracts of *T. terrestris* reduced the viability of a HepG2 cancer cell line (Fig. 4), confirming the previous results of cytotoxic activity^9^ M^31,32^.

In literature, potent co-relationships have been reported between cell cytotoxicity evaluated by the LDH assay and anti-oxidant flavonoids and total phenolic compounds assessed through DPPH assay measuring radical scavenging activity. The main components of *T. terrestris* species have flavonoids and phenylethanoids possessing anti-oxidant activity. Theyare responsible for protecting the cell membrane from oxidative damage, which may improve cell viability and reduce cytotoxicity was later confirmed by the DPPH assay^33,34^ (Fig. 5). In the current study, an improvement in cell survival was detected in the LDH assay (Fig. 6). LDH assay was used to monitor the amount of lysed cells by any damage in the plasma membrane, and the results showed indirect detection of necrosis linked to the leakage of the cytosolic enzyme due to loss of control of membrane permeability. In the case of DPPH assay, the study highlighted the significant anti-oxidant potential of polyphenol-rich *T. terrestris* extract. Through its anti-oxidant capacity, its pretreatment inhibited mitochondrial alterations and necrosis caused by ischemia. Anti-oxidant metabolites inhibit and remedy oxidative stress-related diseases like diabetes, cancer, Alzheimer’s disease, atherosclerosis, and stroke^35,36^. Moreover, the study of the anti-oxidant activity of our plant extract exhibited potent activity to scavenge the free radical DPPH.

Most diseases and disorders are mainly associated with oxidative stress due to free radicals. Free radicals are the base of any biochemical process and represent an essential part of metabolism and aerobic life^37^. Oxidative stress describes the presence of free radicals and reactive oxygen species, which are produced under physiological conditions but become more unsafe and deleterious when not removed by the endogenous system^38^. However, oxidative stress will differentiate endogenous anti-oxidant and reactive oxygen species. ROS results in various diseases like cardiovascular disease, cancer, Alzheimer’s disease, cytotoxic, and causes tissue injuries^39^ Anti-oxidantssupply electrons to the free radicals and thus reduce the adverse effects by stabilizing them. Naturally, anti-oxidants in leafy vegetables and seeds such as vitamin E, Vitamin C, and phenolic compounds can decrease the oxidative damage linked with many diseases, including diabetes, immune-deficient arthritis, cancer, cardiovascular and ageing^40,41^. Phenolics and flavonoids have been reported to hold robust anti-oxidant properties. The anti-oxidant activity of polyphenols helps prevent and cure diseases that are majorly associated with free radicals^42^. In the current study, Methanol and ethanol-basedplant root extractMshowed anti-oxidant activity after 30 min of incubation. Methanol has more inhibition with increasing concentrations up to 80 μg/ml (Fig. 7).

The TNF-α and IL-1β markers were also examined at the protein level using ELISA, which demonstrated a substantial decrease in methanolic plant extract treated groups (Fig. 9). These findings are consistent with earlier studies^43^. The current investigation found a significant drop in TNF-α and IL-1β protein levels, resulting in the downregulation of pro-inflammatory markers, implying that plant extract has an inhibitory influence on these markers.

The methanol extract of *the Tribulusterrestris* plant showed superior activity to its counterpart ethanol-based extract in various assays. As a result, we investigated the gene expression profiling of inflammatory marker genes in the HepG-2 liver cancer cell line after treatment with methanol extract. Among cancer patients, those with diabetes have a higher occurrence of all-cause mortality than those without diabetes. Probable metabolic links between diabetes mellitus (DM) and cancer development are hyperglycemia, hyperinsulinemia, and chronic inflammation.The biochemical process of insulin signaling proceeds by insulin receptor autophosphorylation itself or by directly maintaining the substrate by activating multiple pathways including lipid kinase PI3K/Akt, Bax/Bcl-2, and mTOR leading to carcinogenesis by abnormal cell proliferation and retardation of apoptosis^44,45^.

**Figure 10:**
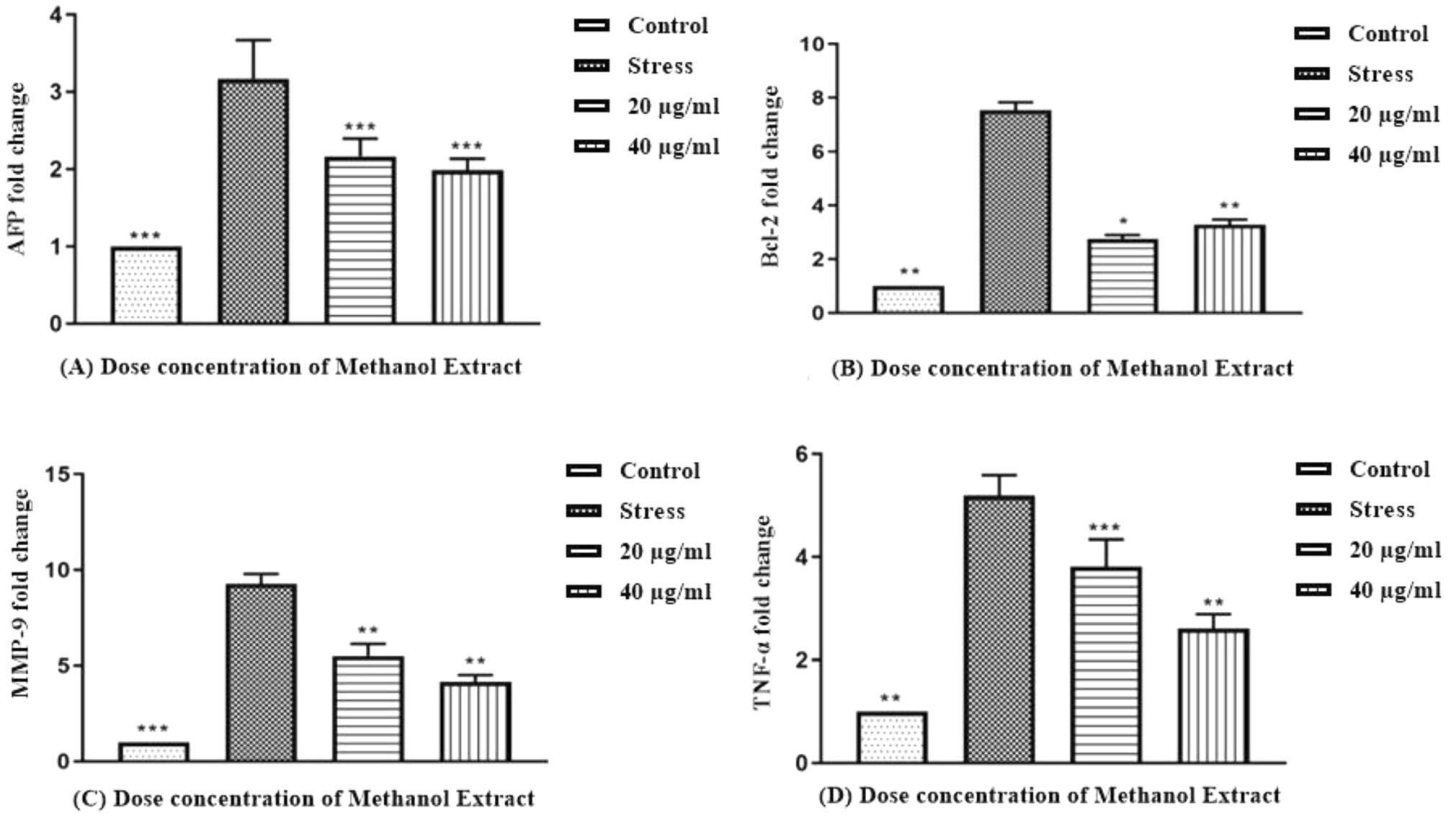
The mRNA expression level of anti-apoptotic and inflammatory markers in HepG-2 liver cancer cell line treated with methanolic plant extract of *T.terrestris*(**a**) indicates mRNA expression of AFP, (**b**) reveals mRNA levels of Bcl-2, (**c**) fold change of MMP-9, (**d**) effect on TNF-α mRNA expression. Bars highlight standard deviation. ∗ , ∗  ∗ and ∗  ∗  ∗ indicate the significance level at *p* < 0.5, *p* < 0:01, *p* < and *p* < 0:001.

IL-1β was used to trigger various inflammatory signal transduction pathways. Therefore, we utilized it to simulate oxidative stress and inflammatory conditions in vitro. It is widely understood that numerous signaling pathways are involved in the inflammatory process, which impairshomeostasis^46^. When methanolic plant extract treated HepG-2 cells were compared to their counterpart IL-1β treated stress group, there was a substantial decrease in mRNA expression of TNF-α, AFP, Bcl-2, and MMP-9 (Fig. 10). MMP-9 (matrix metalloproteinase-13) is a metalloproteinase enzyme involved in the breakdown of the extracellular matrix. MMPs are overexpressed in inflammatory situations. MMP-9, despite its involvement in differentiation, aids in activating TNF pro-inflammatory markers by cleaving pro-TNF into bioactive TNF^47,48^. One study reported that silencing of LPA1 notably attenuated LPA-induced MMP-9 expression and HCC cell invasion. The induction of MMP-9 through coordinated activation of PI3K and p38 MPAK signaling cascades provides novel biomarkers and probable therapeutic targets for HCC.

On the other hand, increased MMP9 activity has been shown to play a role in stimulating endothelial cell apoptosis and dysfunction in diabetic mice. MMP-9 knockout in diabetic mice prominently decreased the nephropathy changes. MMP-9 disrupted podocyte cell integrity, promoting podocyte monolayer permeability to albumin and extracellular matrix protein synthesis. In diabetic patients, urinary MMP-9 concentrations were upregulated before the onset of microalbuminuria. Thus, MMP-9 appears to play a role in the development of diabetic nephropathy^49–51^.

Compared to control cells, methanolic plant extract of *T.terrestris* induced a significant drop in Bcl-2 levels in the current investigation. It implies that the plant extract promotes a pro-apoptotic tendency in HepG2 cells. This activity has been reported in previous studies in the case of MCF-7 breast cancer cell lines^52^. The Bcl-2 gene produces an apoptosis-blocking protein in the mitochondrial membrane. It is over-expressed in a variety of malignancies^53^. High levels of AFP can indicate liver cancer, ovarian or testicular cancer, and non-cancerous liver disorders such as cirrhosis and hepatitis. AFP levels may be raised due to tumor development or regenerated hepatocytes. Many patients with HCC have elevated AFP serum expression levels, and a continuously elevated AFP level is a risk factor for HCC development^54,55^. Many studies have shown that AFP acts as an immune suppressor, promotes malignant transformation during HCC development, and may be involved in the MDR process in patients with liver cancer^56^. In the current study, mRNA expression was significantly downregulated in the HepG-2 cancer cell line after treatment with plant extract.

Previous studies have reported that matrix metalloproteinases can activate IL-1β via proteolytic processing. This study revealed that IL-1β-induced HepG-2 cells exhibited higher MMP-9 expression than their untreated group. Previous research has also shown that MMP-9 is down-regulated in human HNSCC, HN22, HSC-3, and RAW 264.7 cell lines when treated with various plant extracts^57,58^.

TNF-α and IL-1β marker expression levels were also measured to determine gene regulation of these cytokines. IL-1β binds to its receptor leading to the activation of macrophages, intratumoral assembly of immunosuppressive myeloid cells, invasiveness, tumor growth, metastasis, and angiogenesis. This master inflammatory cytokine has also been reported to contribute to the failure of β-cell in the pancreas^46^. Various studies have reported that therapeutic interventions targeting IL-1β have ameliorated DM disease severity, albeit with variegated results in numerous clinical trials. TNF-α is an adipocytokine involved in developing insulin resistance and the pathogenesis of T2DM by disrupting insulin signaling through serine phosphorylation. The binding of TNF-α to TNFRII leads to activation of the NF-κB, JNK, p38 MAPK, ERK, and PI3K pathways. It induces tissue-specific inflammation by association with ROS generation^59^. The literature has reported that necrosis of DEN-exposed hepatocytes activated neighboring myeloid cells in the liver to produce hepatic mitogens that endorsed compensatory explosion of surviving and mutated hepatocytes; TNF-α was one of the significant liver mitogens. One study demonstratedthe deletion of IKKβ in hepatic Kupffer cells, which resulted in decreased TNF-α, IL-6, and HGF and reduced liver tumorigenesis^60,61^. Our findings show a decrease in TNF-α mRNA expression and protein levels of TNF-α and IL-1β in *T.terrestris* treated methanolic plant extract. Likewise, down-regulation of IL-1β and TNF-α at the mRNA and protein levels has recently been described in Lipopolysaccharide-Induced Human Monocyte-derived Macrophagesyy^62,63^. Therefore, we can conclude that inhibiting these molecular moieties can dampen the progression of T2DM and malignancy. In this regard, *T. terrestris* plant extract exhibited robust anti-diabetic and cytotoxic activity activities, which can bestow potent drug candidates.

## Conclusion

The current study reports anti-diabetic, anti-inflammatory, anti-oxidant, and anti-proliferative properties of *T. terrestris* plant extract treated HepG2 liver cancer cell line. *T. terrestris*methanolic plant extract alleviated the adverse effects of IL-1β induced stress in vitro via activating the inflammatory pathways. The methanolic plant extract of *T. terrestris* reduced the expression of inflammatory mediators and cytokines in a dose-dependent manner, which has a favorable effect on various inflammatory disorders, including diabetes and cancer. The findings could help with the therapeutic applications *T.terrestris* by identifying potential drug metabolites to treat inflammatory diseases.

## Data Availability

All data generated or analyzed during this study are included in the manuscript.
